# Hot-Melt Extrusion of the Thermo-Sensitive Peptidomimetic Drug Enalapril Maleate

**DOI:** 10.3390/pharmaceutics14102091

**Published:** 2022-09-30

**Authors:** Lena Hoffmann, Jörg Breitkreutz, Julian Quodbach

**Affiliations:** 1 Institute of Pharmaceutics and Biopharmaceutics, Heinrich Heine University, Universitätsstraße 1, 40225 Düsseldorf, Germany; 2 Department of Pharmaceutics, Utrecht University, Universiteitsweg 99, 3584 CG Utrecht, The Netherlands

**Keywords:** hot-melt extrusion, peptidomimetic drug, thermal degradation, analytics of extrudates, HPLC method development, content uniformity, personalized medicine, treatment of hypertension and heart failure

## Abstract

The aim of this research was the production of extrudates for the treatment of hypertension and heart failure and the investigation of the degradation of the peptidomimetic drug enalapril maleate (EM) during hot-melt extrusion (HME). A fast HPLC method was developed to quantify enalapril maleate and possible degradation products. Screening experiments revealed that the diketopiperazine derivative (Impurity D) was the main degradation product. Hot-melt extrusion of enalapril maleate with the polymer Soluplus^®^ enabled extrusion at 100 °C, whereas a formulation with the polymer Eudragit^®^ E PO could be extruded at only 70 °C. Extrusion at 70 °C prevented thermal degradation. A stabilizing molecular interaction between enalapril maleate and Eudragit^®^ E PO was identified via FT-IR spectroscopy. Dissolution studies were carried out to study the influence of the formulation on the dissolution behavior of enalapril maleate. These promising results can be transferred to other thermo-sensitive and peptidomimetic drugs to produce extrudates which can be used, for instance, as feedstock material for the production of patient-specific dosage forms via Fused Deposition Modeling (FDM) 3D printing.

## 1. Introduction

**Figure 1:**
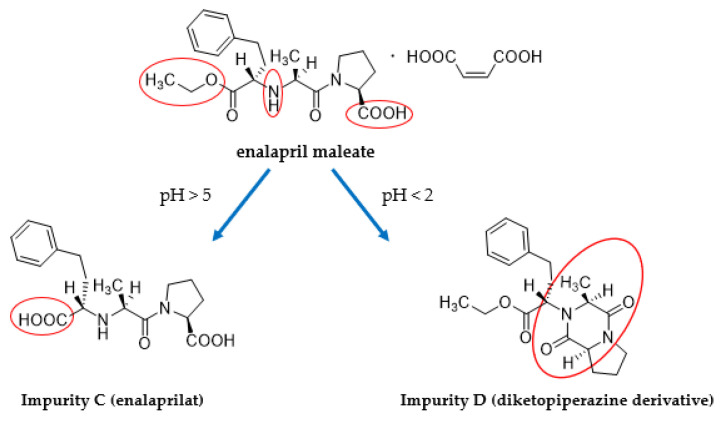
Illustration of the main degradation products of enalapril maleate [5].

The peptidomimetic drug enalapril maleate is an angiotensin-converting enzyme inhibitor (ACEI), which is one of the active ingredients on the World Health Organization (WHO) model list of essential medicines and is used, in particular, for heart failure and hypertension in both adults and children [1,2]. Enalapril maleate is the prodrug of the active metabolite enalaprilat and has a similar structure to the tripeptide phenylalanine (Phe)-alanine (Ala)-proline (Pro) [3]. The active pharmaceutical ingredient belongs to the biopharmaceutics classification system (BCS) class III with high solubility and low permeability [4]. In an aqueous solution, two main degradation products, enalaprilat and a diketopiperazine derivative (DKP), have been identified which are described in the European Pharmacopoeia as Impurities C (Imp-C) and D (Imp-D) of the starting material besides the other Pharmacopoeial Impurities. The rate and pathways of the degradation are pH-dependent. Below pH 2, the main degradation product is DKP and above pH 5, the main degradation product is enalaprilat (Figure 1) [5]. Whereas the formation of DKP is an intramolecular cyclization typically observed for peptides, the formation of enalaprilat is a hydrolysis reaction [4,6].

Due to the instability of peptidomimetic drugs in an aqueous solution, hot-melt extrusion (HME) can be a preferred method for the preparation of solid dispersions because of the lack of solvents [7,8,9,10].

HME is frequently used for the production of formulations with desired release characteristics, for the taste masking of bitter-tasting drugs and especially for solubility enhancement of poorly soluble drugs [8,9,10,11,12,13,14,15]. Furthermore, HME is a continuous process, which can be easily scaled up [16]. Nevertheless, HME also shows some drawbacks. Thermally unstable drugs may degrade at elevated temperatures and under shear forces present during hot-melt extrusion [17,18]. Therefore, attempts were made to overcome this problem, which also refer to the improvement of solubility or bioavailability of the model drug. Liu et al. (2013) prepared solid dispersions with the thermally unstable drug carbamazepine and used polymer combinations of Kollidon^®^ VA 64 (Ludwigshafen, Germany), Soluplus^®^ (Ludwigshafen, Germany) and Eudragit E^®^ PO (Essen, Germany) as carriers to improve the drug-polymer miscibility and decrease the process temperature [8]. Huang et al. (2017) prepared amorphous solid dispersions of the thermally labile drug gliclazide with the polymer hydroxypropyl methyl cellulose (HPMC) and investigated the degradation kinetics depending on the different polymorphs of gliclazide. From the study, the authors concluded that the degradation of the drug could be influenced by the applied temperature, the unstable nature of the amorphous form of the drug and the mechanical energy input. Optimization of these parameters led to an improved recovery rate of the drug [19]. DiNunzio et al. (2010) produced solid dispersions containing the heat-sensitive active ingredient hydrocortison by hot-melt extrusion and Kinetisol^®^ dispersing. The authors demonstrated that the choice of a suitable carrier for processing at lower temperatures and a reduced residence time could improve product potency [18]. Huang et al. (2019) worked with the heat-sensitive and high-melting drug tadalafil and tried to inhibit the recrystallization of produced amorphous solid dispersions to improve bioavailability [20]; whereas, Kulkarni et al. (2018) applied hot-melt extrusion for the improvement of the bioavailability of the thermolabile drug artemisinin. The degradation could be reduced by producing a solid dispersion with the polymer Soluplus^®^ and the addition of 5% citric acid [21].

The focus of this work was to investigate and quantify the chemical degradation of the model peptidomimetic drug enalapril maleate during hot-melt extrusion in the absence of water. Further, formulations should be developed that enable extrusion with reduced or no degradation. Different polymers were screened for their suitability and the most promising formulations were further optimized. Not only the content of enalapril in the extrudates, but also the dissolution behavior for the optimized formulations was investigated.

## 2. Materials and Methods

### 2.1. Materials

Enalapril maleate was purchased from Azelis (Sankt Augustin, Germany) ex Zhejiang Huahai Pharmaceutical Industry Co. (Zhejiang, China). Hypromellose (HPMC, AFFINISOL™ HPMC HME 15 LV) and POLYOX™ WSR N10 (PEO, Mw 100,000) were kindly provided by DuPont Nutrition & Biosciences (Neu-Isenburg, Germany). Kollidon^®^ 12 PF (K 12 PF), Kollidon^®^ VA 64 fine (K VA 64 fine) and Soluplus^®^ (SOL) were kindly provided by BASF (Ludwigshafen, Germany). Basic butylated methacrylate copolymer (bPMMA, Eudragit^®^ E PO) and fumed silica (SiO_2_, Aerosil 200 V/V Pharma) were kindly provided by Evonik (Essen, Germany). Polyethylene glycol (PEG) 6.000 (Polyglykol^®^ 6000 P) was kindly provided by Clariant (Frankfurt, Germany). Enalapril maleate chemical reference standard (CRS), enalapril for system suitability CRS (containing EM and Impurity A), enalaprilat dihydrate CRS, enalapril impurity G CRS and enalapril impurity mixture A CRS (containing Impurity C and Impurity H) (all European Pharmacopoeia Reference Standards) were purchased at the European Directorate for the Quality of Medicine & Healthcare (Strasbourg, France). Enalapril maleate United States Pharmacopoeia (USP) Reference Standard wase purchased by Eurofins PHAST GmbH (Homburg, Germany). Impurity B and enalapril diketopiperazine were purchased from LGC Standards GmbH (Wesel, Germany). Other chemicals such as solvents and buffering materials were of reagent grade.

### 2.2. Screening Experiments for HME

For the polymer screening, six different formulations with a drug load of 10% enalapril maleate were investigated (Table 1).

To destroy and remove agglomerates, enalapril maleate and the polymers were separately sieved and mixed for 15 min in a turbula mixer (T2F, Willy A. Bachofen, Switzerland).

The powder blends of each formulation were fed with a flat-bottom powder feeder (ZD 5 FB, Three-Tec, Seon, Switzerland) and dosed at a feed rate of 50 g/h or 100 g/h into a co-rotating twin screw extruder Leistritz ZSE12 HP-PH extruder (Leistritz, Nürnberg, Germany) with a screw diameter of 12 mm and a screw length to diameter ratio of 40:1. Screws with two kneading zones and a die with a diameter of 2 mm were used. Different temperatures and process parameters as detailed in Table 2 were applied for the formulations.

### 2.3. Formulations for HME at Reduced Temperatures

HME was carried out again with formulations F5 and F6 for the production of extrudates at reduced temperatures.

Both formulations were fed either with a flat-bottom powder feeder (ZD 5 FB, Three-Tec, Seon, Switzerland) or a volumetric feeder (Brabender MT-S-HYD, Brabender, Duisburg, Germany) at a feed rate of 100 g/h into a co-rotating twin screw extruder (ZSE12 HP-PH, Leistritz, Germany) with a screw diameter of 12 mm and a screw length to diameter ratio of 40:1. Screws with two kneading zones and a die with a diameter of 2 mm were used. The temperature profiles at a constant screw speed of 25 rpm are shown in Table 3.

Following extrusion, the extruded strand was cooled and transported with a conveyor belt (model 846102.001, Brabender, Germany). The desired diameter of the extrudates of 1.75 mm was achieved using a belt haul-off unit of a winder (Brabender, Duisburg, Germany). Extrudates were collected over the whole extrusion process and used for the content determination.

### 2.4. Particle Size Distribution

The particle size distributions were determined via laser diffraction (Mastersizer 3000, Malvern Instruments, Malvern, UK) with dry dispersion using Fraunhofer approximation for data evaluation (*n* = 3). The dispersion pressure was adjusted to 0.8 bar.

### 2.5. Scanning Electron Microscopy (SEM) Imaging

Morphology of both powder mixtures and extrudates were examined using Zeiss scanning electron microscope Leo 1430 VP (Zeiss, Germany). Samples were sputter coated with a thin gold layer. The working voltage ranged from 5 to 10 kV.

### 2.6. Thermal Analysis

Thermo analysis of enalapril maleate starting material and the reference standards of the degradation products were performed using dynamic scanning calorimetry (DSC, DSC 1, Mettler-Toledo, Giessen, Germany). Samples were heated at 10 °C/min from 20 °C to 200 °C. For thermogravimetric analysis (TGA) and derivative thermogravimetric analysis (DTG), enalapril maleate was measured using a NETZSCH TG 209F1 Libra (NETZSCH, Selb, Germany). The sample was placed in an 85 µL aluminium pan and was then heated from 35 °C to 500 °C using 10 °C/min as a heating rate. The thermal decomposition was analyzed using NETZSCH Proteus Software. The experiments were carried out under a nitrogen gas flow of 20 mL/min.

### 2.7. Drug Content of Extrudates

#### 2.7.1. Screening Experiments

Sections of drug-loaded extrudates (approximately 0.1 g), with the exception of formulation F6, were placed in a 100 mL volumetric flask and dissolved in 100 mL 0.1 N hydrochloric acid. Samples of the solutions were then filtered through a 0.20 μm polypropylene filter. The content of enalapril (ENP) and degradation products in the extrudates were determined by high-performance liquid chromatography (HPLC) analysis. An Elite LaChrom system consisting of an L-2200 automatic sampler, L-2130 high pressure pump, L-2300 column oven and L-2400 UV detector was used (all Hitachi-VWR). A Eurospher II 100-5 C18A column (125 mm × 4.6 mm, 5 µm) with integrated precolumn (Knauer, Germany) served as the stationary phase. The mobile phase was composed of acetonitrile and 1 mM potassium dihydrogen phosphate buffer (pH 3.0, adjusted with orthophosphoric acid 85%). The flow rate was 1 mL/min and the column temperature was 50 °C. Detection was performed at a wavelength of 215 nm. After injection of 10 µL of the sample solution samples were separated under gradient conditions (Table 4).

For formulation F6, a section of a drug-loaded extrudate (approximately 0.1 g) was placed in a 100 mL volumetric flask and was dissolved in 100 mL of a mixture of acetonitrile and 1 mM potassium dihydrogen phosphate buffer pH 3.0 (50/50, *v*/*v*). Samples of the solutions were then filtered through a 0.45 μm nylon filter. The content of enalapril and degradation products in the extrudates were determined by HPLC analysis with an optimized gradient (Table 5).

#### 2.7.2. Samples from Extrusion at Reduced Temperatures

The HPLC method with the gradient shown in Table 5 was further used for the quantification of the drug content of formulations F5 extruded at optimized conditions and F6.

Extrudate samples were collected and analyzed at the beginning, the middle and end of extrusion (*n* = 10, mean ± SD). Therefore, sections of a drug-loaded extrudate (approximately 0.1 g) were placed in a 100 mL volumetric flask and dissolved in 100 mL of a mixture of acetonitrile and 1 mM potassium dihydrogen phosphate buffer pH 3.0 (50/50, *v*/*v*). Samples of the solutions were then filtered through a 0.45 μm nylon filter. An Elite LaChrom system (VWR, Darmstadt, Germany) consisting of an L-2200 automatic sampler, L-2130 high pressure pump, L-2300 column oven and L-2400 UV detector was used.

**Figure 2:**
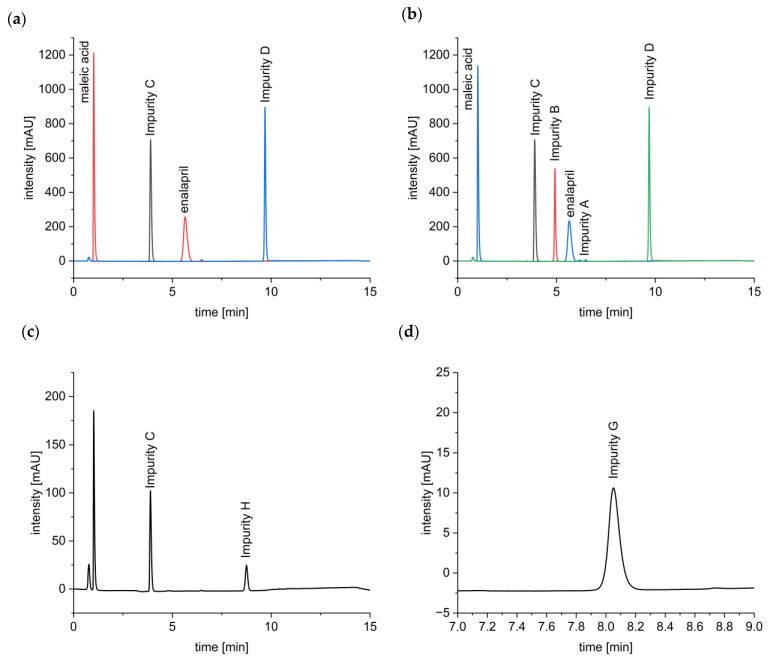
HPLC chromatograms of EM and the main degradation products Imp-C and Imp-D (**a**), of EM and Imp-A, Imp-B, Imp-C and Imp-D (**b**), of Imp-C and Imp-H (**c**) and of Imp-G (**d**).

Compared to the originally developed method and, also, already published methods, the separation of enalapril and possible degradation products with the optimized gradient was carried out on an XBridge C18 column (3.0 × 150 mm, 3.5 µm) at a temperature of 65 °C and an injection volume of 30 µL [22,23]. The temperature increase leads to an improved peak shape of enalapril [24] and a higher separation efficacy between enalapril and pharmacopoeial Impurity A. Furthermore, the method allows on the one hand the complete separation of maleic acid and enalaprilate (Imp-C), which makes the quantification of this possible main degradation product more reliable (Figure 2a). On the other hand, the separation of enalapril and the known pharmacopoeial impurities A, B, C, D, G and H is possible in a single run time of 15 min (Figure 2b–d).

The identification of the related substances was done with solutions of each standard. For the selectivity of the developed HPLC method, the enalapril maleate CRS solution was spiked with the other related substances. The determination of the limit of detection (LOD) and the limit of quantification (LOQ) for enalapril resulted in concentrations of 19.4 ng/mL and 58.7 ng/mL. Linearity was given for the content uniformity of enalapril in the extrudates in the concentration range of 60 to 140 µg/mL and for the dissolution in a concentration range of 0.2 to 12 µg/mL with a correlation coefficient of R^2^ > 0.999. The accuracy of the content in the samples was ensured by using the enalapril maleate USP reference standard and enalapril diketopiperazine standard with a known content. The calibration for enalapril diketopiperazine was always performed with the external standard. Precision was determined in terms of repeatability for enalapril, where the coefficient of variation (CV) was 0.74%.

### 2.8. FT-IR Spectra Measurements

FT-IR spectra measurements were made to investigate possible interactions between enalapril maleate and the polymers. Therefore, infrared spectra were recorded with a Shimadzu IR Affinity-1 with ATR sampling technique (Shimadzu, Duisburg, Germany).

### 2.9. Dissolution

In vitro drug release studies were carried out using dissolution tester AT7 Smart (Sotax, Aesch, Switzerland) with USP type I apparatus (basket apparatus) at 37 °C ± 0.5 °C with a rotating speed of 50 rpm in 900 mL phosphate buffer at pH 6.8 or in 0.1 N hydrochloric acid. Extrudates were taken from the lowest possible extrusion temperature. The amount of EM was quantified by HPLC.

## 3. Results and Discussion

### 3.1. Raw Material Properties

#### 3.1.1. Particle Size Distribution

The determination of the median particle size x50 of the raw materials showed large differences between the polymers bPMMA and SOL (Table 6). The median particle size of bPMMA was 9.7 µm ± 0.1 µm and 308 µm ± 6.1 µm for SOL. PEO had a median particle size of 111 ± 8.6. In contrast, the median particle size of the active ingredient enalapril maleate was 47.2 ± 1.3 µm.

#### 3.1.2. Scanning Electron Microscopy (SEM) Imaging

**Figure 3:**
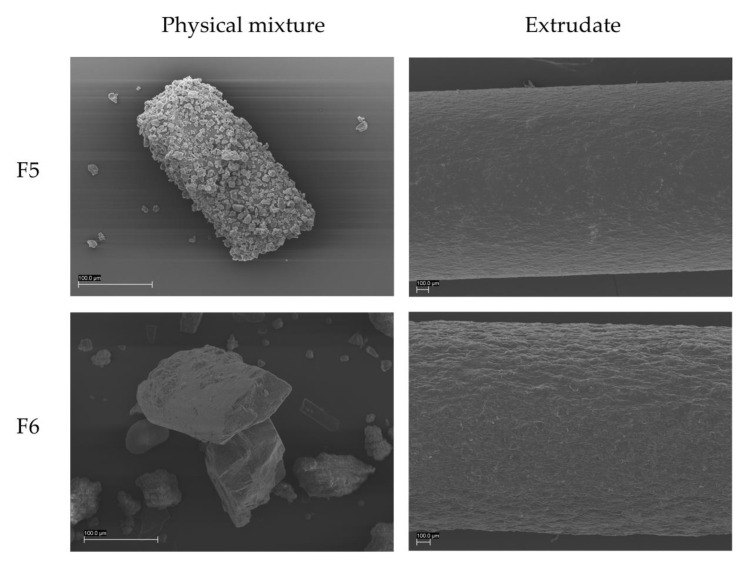
SEM images of the physical mixtures and extrudates of formulation 5 and 6 (Scale: 100 µm).

SEM images showed that enalapril maleate was present in crystalline form as platelets in both physical mixtures. In formulation F5, the small particles of bPMMA adhered to the larger particles of enalapril maleate, resembling a dry coating. Formulation F6 does not display any kind of interaction between SOL and enalapril maleate. In the SEM images of both extrudates, crystalline parts of the semi-crystalline PEO could be observed on the surface, as previously described by Tidau et al. (2019) for PEO-based extrudates [25] (Figure 3).

#### 3.1.3. Thermal Analysis

**Figure 4:**
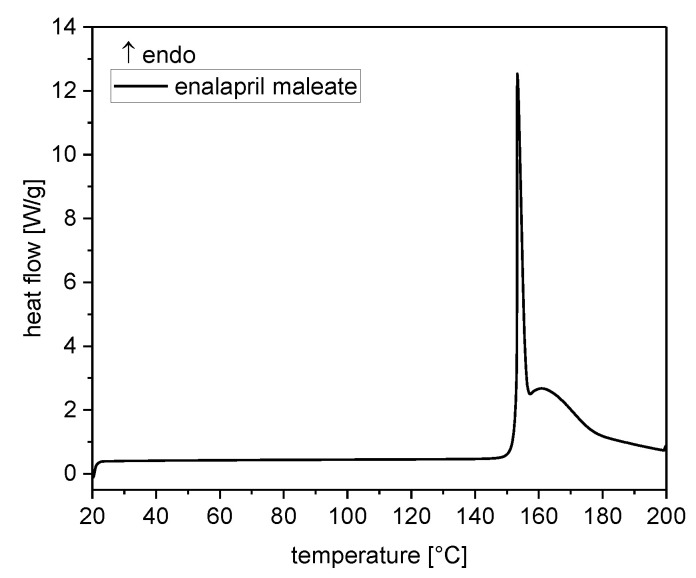
DSC thermogram of enalapril maleate starting material.

**Figure 5:**
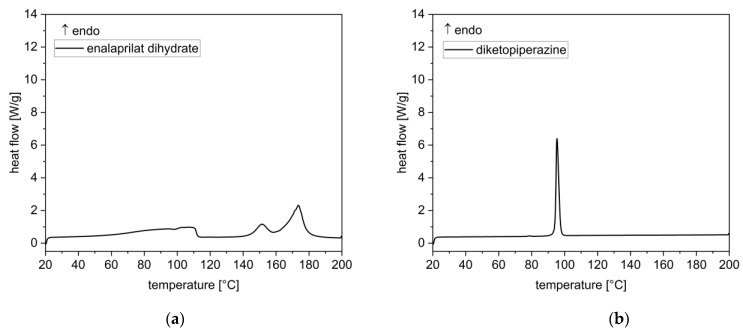
DSC thermograms of the main degradation products enalaprilat as dihydrate (**a**) and enalapril diketopiperazine derivative (**b**).

DSC analysis of enalapril maleate starting material showed two superimposed peaks in the range from 150 °C to 200 °C, as previously described by Lin et al. (2002) [26,27]. The intense and sharp peak at approximately 153 °C was caused by melting and the broad thermal event at 163 °C was caused by thermal decomposition (Figure 4). The degradation product enalaprilat showed an endothermic peak at 151 °C and a second peak at 173 °C, whereby the second thermal event started at 165 °C (Figure 5a). The thermogram of the degradation product DKP showed a minimal first endothermic event at 78 °C and a second endothermic peak at 95 °C, which was associated with the melting of the substance (Figure 5b).

**Figure 6:**
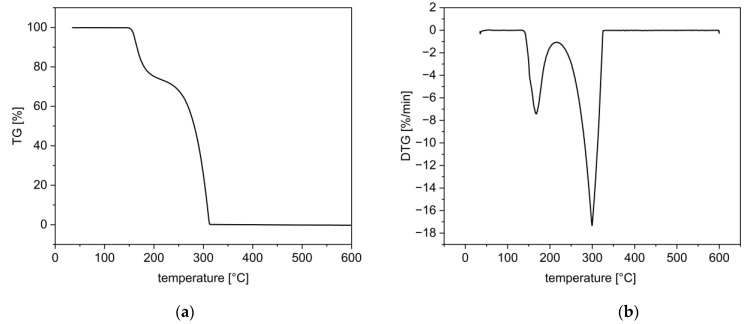
TG (**a**) and DTG curves (**b**) of enalapril maleate.

Thermogravimetric analysis (TGA) confirmed the decomposition of enalapril maleate as observed in the DSC data. TGA showed an onset at a temperature of approximately 154 °C and a mass loss of approximately 27% up to a temperature of 220 °C (Figure 6a) [27,28]. The derivative thermogravimetric curve showed the maximum of the initial decomposition at a temperature of approximately 168 °C (Figure 6b).

Consequently, extrusion must take place below the melting temperature of enalapril maleate, since melting was accompanied by decomposition.

### 3.2. Polymer Selection for HME

#### 3.2.1. Screening Experiments

Obtained extrudates from screening experiments differed in their properties. Formulation F1 with the polymer K 12 PF could be extruded between 120 °C and 130 °C. While extrudates at 120 °C exhibited a rough surface, likely due to the glass transition temperature (Tg) of K 12 PF at 90 °C being close to the extrusion temperature [29,30], extrudates processed at a temperature of 130 °C showed a smooth surface and were collected for further analysis.

Formulation F2 with the polymers HPMC and bPMMA were extruded at 130 °C because the polymer HPMC has a higher Tg of 115 °C compared to the polymer bPMMA with a lower Tg of 57 °C [31,32,33]. Extrudates from formulation F2 appeared orange and had a smooth surface.

Formulation F3 with the polymers K 12 PF and K VA 64 could be extruded at a temperature of 140 °C as the lowest extrusion temperature. The reason for this is the higher glass transition temperature of the polymer K VA 64 with a Tg of 101 °C [29,30]. Extrusion of both formulations was not possible at lower temperatures, although Kempin et al., (2018) and Kollamaram et al., (2018) could extrude these polymers below 100 °C with a self-constructed extruder [34] and with a single screw extruder [35].

Extrudates from formulations F1 and F3, which contained either K 12 PF alone or a combination of K 12 PF and K VA 64 appeared milky-white and sticky. The extrudates of both formulations were smooth, but brittle despite a high plasticizer content.

Formulation F4 with the polymer HPMC could be extruded at a temperature of 140 °C, which is considered a suitable process temperature for HPMC in literature and can be explained by the higher proportion of this polymer in the formulation [25,32,36,37,38,39]. The extrudates from formulation F4 appeared orange similar to formulation F2. This could be explained by the formation of the diketopiperazine derivative. The extrudates with HPMC had a rough surface in comparison to the smooth surface of the extrudates of formulation F2.

Formulation F5 and F6, unlike the previous formulations, were extruded at a feed rate of 100 g/h.

Compared to 50 g/h, 100g/h was used to investigate the influence of the feed rate on the degradation of enalapril maleate. Formulation F5 was extruded at a temperature above the melting point of enalapril maleate at 150 °C [40], whereas formulation F6 was extruded at a temperature of 100 °C. The extrudates of F5 appeared yellowish and the extrudates of F6 slightly yellowish. Both filaments had a smooth surface.

#### 3.2.2. Optimized Process Conditions

Due to their low glass transition temperatures, bPMMA (Tg = 57 °C) and SOL (Tg = 70 °C) in formulations F5 and F6 were identified as suitable formulations for extrusion at lower temperatures [33,41]. The extrusion of F5 with the polymers bPMMA and PEO was performed under optimized conditions at temperatures of 70 °C and 100 °C [7], whereas the extrusion of F6 with the two polymers SOL and PEO was repeated at a temperature of 100 °C, identified as lowest possible process temperature. Lightly yellowish-colored extrudates obtained from the extrusion process had a smooth surface and were flexible.

### 3.3. Drug Content of Extrudates

#### 3.3.1. Content Uniformity of the Extrudates of the Screening Experiments

**Figure 7:**
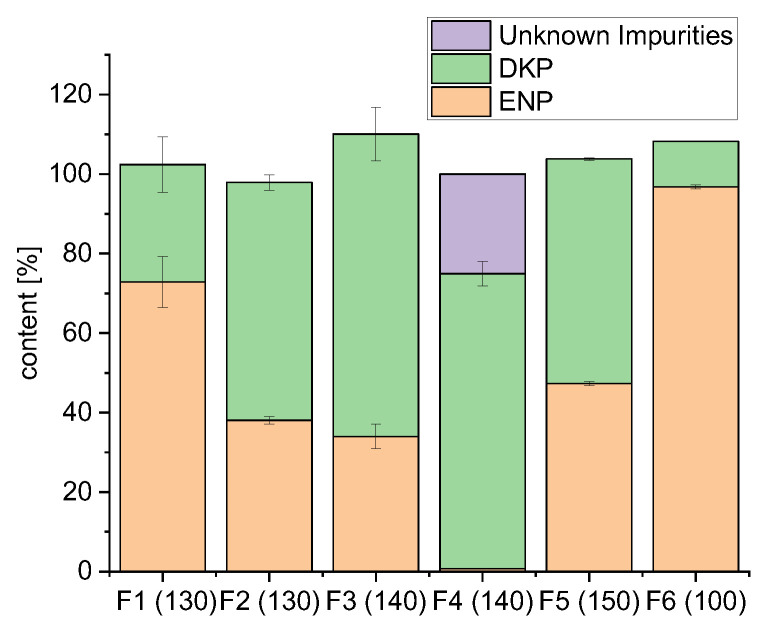
Content uniformity of the extrudates of the screening experiments (*n* = 6, mean ± SD).

The results of the content uniformity showed that in all formulations, besides the active ingredient enalapril maleate, the diketopiperazine derivative (DKP, Imp-D) occurred as the main thermal degradation product in the extrudates (Figure 7). Formulation F1 with K 12 PF showed the highest content of enalapril. In the extrudates, 72.88 ± 6.42% enalapril was found, whereas 29.50 ± 6.97% Imp-D were determined. Formulation F2 with the polymers HPMC and bPMMA also extruded at a temperature of 130 °C showed a higher degradation. 38.05 ± 1.00% enalapril and 59.84 ± 1.98% Imp-D were recovered. Formulation F3 with the polymers K 12 PF and K VA 64 was extruded at 140 °C and showed a further decrease in the content of enalapril. 34.01 ± 3.09% enalapril and 76.05 ± 6.74% Imp-D were determined. Formulation F4 with the polymer HPMC showed an almost complete degradation of enalapril and only 0.74 ± 0.10% was found. 74.18 ± 3.05% Imp-D and further unidentified impurities could be detected.

From the extrusion of these four formulations, which were all fed at a feed rate of 50 g/h, it could be observed that not only the temperature but also the used polymers have an influence on enalapril maleate degradation. The formulations F2 and F4, which both contained the polymer HPMC showed, due to a higher melt viscosity, a higher pressure at the die during extrusion [37,42]. This could explain the higher amount of degradation of enalapril in formulation F2 and especially in formulation F4 (Figure 7), which was also observed during hot-melt extrusion of gliclazide with HPMC in a previous publication by Huang et al. (2017) [19]. Formulation F5 was fed with the higher feed rate of 100 g/h into the extruder and the extrusion was performed at a temperature of 150 °C. In the extrudates, 47.33 ± 0.50% enalapril and 56.44 ± 0.34% Imp-D were determined. The results showed that also the feed rate had an impact on the degradation. Despite the higher extrusion temperature of 150 °C, more enalapril was found compared to formulations F2, F3 and F4. For formulation F6 with the polymer SOL and PEO, which was also fed with a feed rate of 100 g/h and extruded at a temperature of 100 °C, the highest amount of enalapril could be found. 96.78 ± 0.54% enalapril was recovered and 11.43 ± 0.05% Imp-D.

The process temperature and also the applied shear forces had a great impact on enalapril maleate degradation. A higher feed rate could shorten the residence time, and thus, reduce the amount of degradation. Process conditions and formulations were optimized since degradation of enalapril maleate already takes place below the melting temperature contrary to DSC and TG measurements. The optimized formulations F5 and F6 containing the polymers bPMMA and SOL were extruded again at reduced temperatures and with a higher feed rate of 100 g/h into the extruder.

#### 3.3.2. Content Uniformity for the Formulations under Optimized Conditions

**Figure 8:**
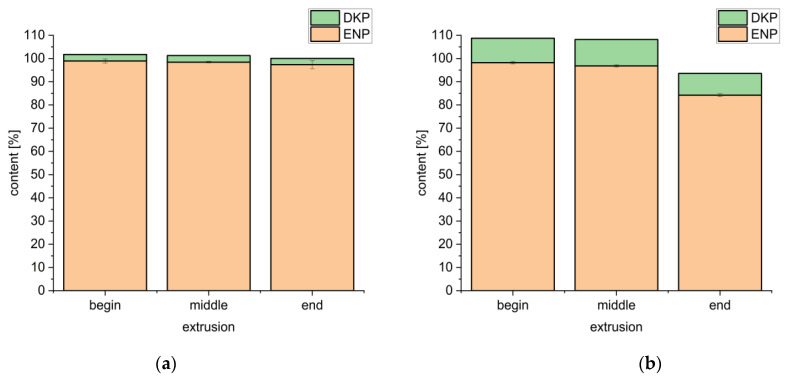
Enalapril and diketopiperazine derivative contents over the extrusion processes at 100 °C of formulation 5 (**a**) and formulation 6 (**b**) (*n* = 10, mean ± SD).

For formulation F5 with bPMMA extruded at 100 °C, it was observed that the content of enalapril remained constant over the extrusion and was 98.44 ± 0.30% versus 2.83 ± 0.09% Imp-D in the middle of the run (Figure 8a). For formulation F6 with the polymer SOL also extruded at 100 °C it was observed that the content of enalapril decreased to a greater degree during extrusion. In the middle, the content of enalapril was 94.83 ± 1.72% and 7.54 ± 0.24% Imp-D (Figure 8b). The larger decrease in the content of enalapril could be due to an inhomogeneous powder mixture which could be explained by a larger particle size of SOL (30 ± 6.1 µm) compared to bPMMA (9.7 ± 0.1 µm). The ×50 of enalapril maleate is 47.2 ± 1.3 µm. By comparing the two formulations, a lower content was found for formulation F6 under the same process conditions.

**Figure 9:**
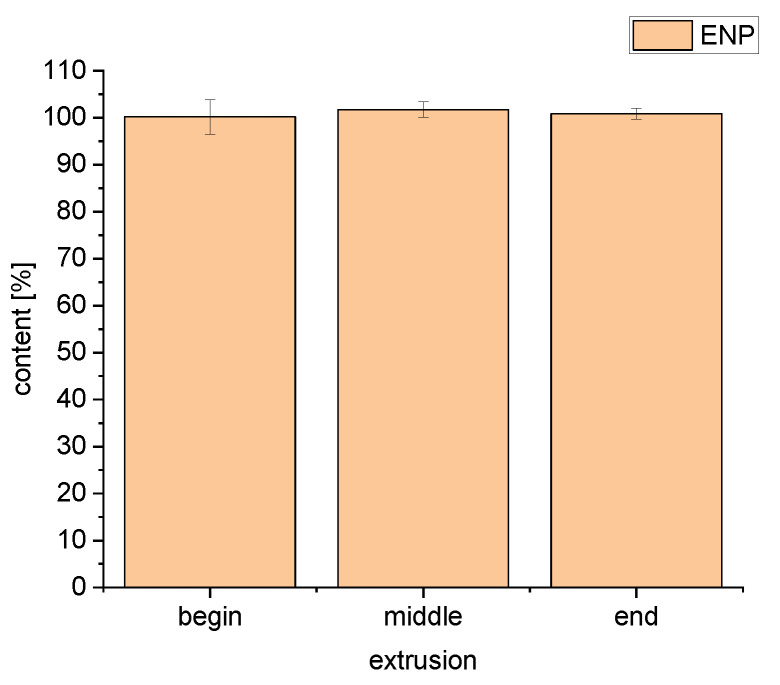
Content uniformity over the entire extrusion for formulation 5 at 70 °C (*n* = 10, mean ± SD).

The lowest possible extrusion temperature for F6 was 100 °C, whereas F5 could be extruded at 70 °C. The content of enalapril was 101.72 ± 1.65% and no degradation products were found (Figure 9). The different recovered enalapril concentrations after extrusion at 100 °C imply a potentially stabilizing interaction between enalapril malate and bPMMA.

### 3.4. FT-IR Spectra Measurements

**Figure 10:**
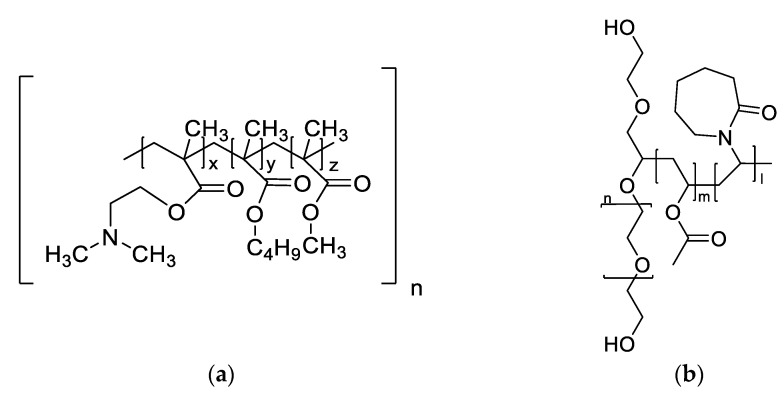
Structural formulas of bPMMA (**a**) and SOL (**b**) [41,45].

FT-IR spectroscopy was used to investigate interactions between enalapril maleate and the polymers. The structures of the basic polymer bPMMA and the neutral polymer SOL are shown in Figure 10.

The FT-IR spectrum of enalapril maleate showed characteristic bands at 1749 cm^−1^ (C=O of the carboxylic group), 1645 cm^−1^ (C=O of the tertiary amide), 1578 cm^−1^ (C=O of the carboxylic acid of monohydrogen maleate) and 1225 cm^−1^ (C-C-O of acetate and ester) [43,44]. Characteristic bands for the dimethylamino groups of bPMMA could be found at 2822 cm^−1^ and 2770 cm^−1^. FT-IR spectrum of bPMMA showed further characteristic peaks for the ester groups at 1144 cm^−1^, 1238 cm^−1^ and 1267 cm^−1^ and the C = O ester vibration at 1724 cm^−1^ [43,45]. FT-IR spectrum of SOL showed characteristic bands at 2922 cm^−1^ (C-H), 2857 cm^−1^ (C-H), 1740 cm^−1^ (C=O of the carboxylic group) and 1633 cm^−1^ (C=O of the amide group) [46,47]. FT-IR spectrum of PEO showed characteristic bands at 2888 cm^−1^ (C-H), 1240 cm^−1^ (O-H), 1093 cm^−1^ (C-O) and 1059 cm^−1^ (C-O) [48].

**Figure 11:**
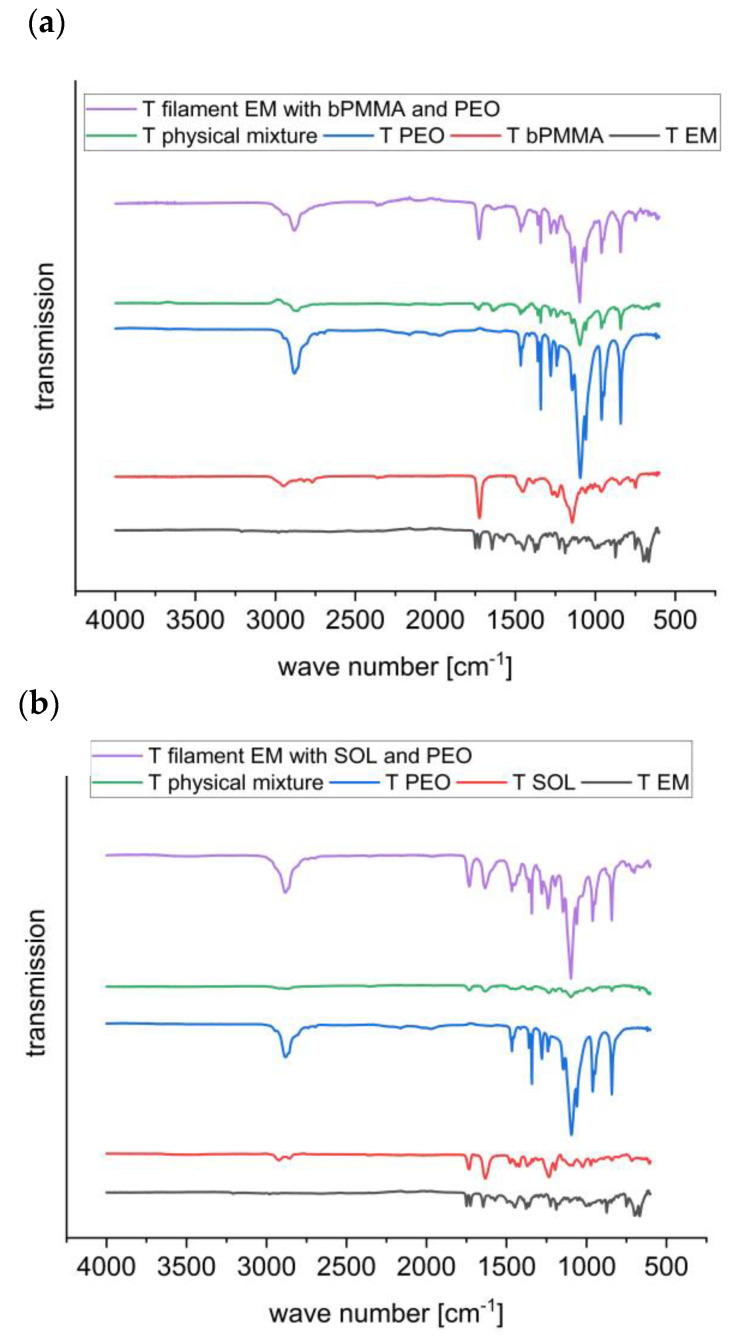
FT-IR spectra for formulations 5 (**a**) and 6 (**b**).

For the filament of F6 containing the polymer SOL no interaction could be observed as peak shifts or loss of disappearance of peaks. (Figure 11b), whereas for the filament of formulation F5 containing the polymer bPMMA differences in the spectra could be detected (Figure 11a).

For formulation F5, the absence of characteristic bands for the spectrum of enalapril maleate at 1740 cm^−1^ and 1578 cm^−1^, and also, for dimethylamino groups of the bPMMA at 2822 cm^−1^ and 2770 cm^−1^ indicates a cation-anion interaction between the carboxylic groups of enalapril maleate and the dimethylamino groups of bPMMA (Figure 11a) [7,49]. Wang et al. (2004) investigated this phenomenon in casted films with enalapril maleate and bPMMA and postulated that one carboxylic group of maleic acid could interact with the amino group of enalapril and the other carboxylic group of maleic acid and/or the carboxylic group of enalapril could interact with the dimethylamino groups of bPMMA [50,51]. This effect could explain the stabilizing effect when enalapril maleate is hot-melt extruded with bPMMA as a polymer [52]. No changes were observed in the spectra due to the use of PEO.

### 3.5. Dissolution

The release profile of enalapril from the extrudates containing SOL and bPMMA was also investigated.

The US Pharmacopeia (USP) monograph for enalapril maleate tablets specifies that at least 80% enalapril has to be released after 30 min in the paddle apparatus (USP II method) in phosphate buffer pH 6.8 at a rotational speed of 50 rpm [53]. However, during previous dissolution studies, it was observed that extrudates wrapped around the paddle and this phenomenon influenced the dissolution behavior. Therefore, investigations were made with the basket apparatus (USP I method).

**Figure 12:**
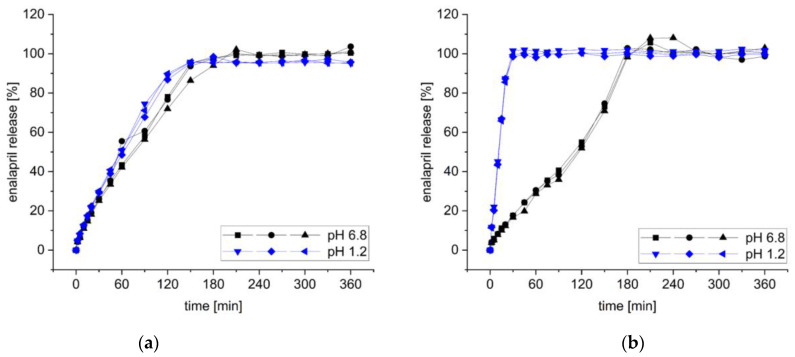
Dissolution behaviour of extrudates with SOL (**a**) and bPMMA (**b**) in basket apparatus in phosphate buffer pH 6.8 and 0.1 N hydrochloric acid (*n* = 3).

In the basket apparatus formulation F6 with SOL released 25.81 ± 0.53% enalapril after 30 min and 91.74 ± 4.72% after 150 min (Figure 12a). After 180 min 96.44 ± 2.16% enalapril was released. Alongside the dissolution of extrudates with SOL in phosphate buffer pH 6.8, the dissolution behavior of enalapril in 0.1 N hydrochloric acid in the basket apparatus was investigated. Of enalapril, 29.52 ± 0.57% was released after 30 min and 88.53 ± 1.60% enalapril was released after 120 min and after 180 min 96.55 ± 1.73% enalapril was released. In comparison to the dissolution in phosphate buffer pH 6.8 the criterion of Q = 80% is reached earlier but, nevertheless, after 180 min approximately the same amount of enalapril is released (96.44 ± 2.16% vs. 96.55 ± 1.73%) (Figure 12a). These results indicate the pH-independent dissolution of enalapril from the extrudates with SOL.

Formulation F5 with bPMMA extruded at 70 °C released 17.39 ± 0.57% after 30 min and 100% after 180 min in phosphate buffer pH 6.8 (100.19 ± 2.47%). However, when released in 0.1 N hydrochloric acid, 99.87 ± 1.56% enalapril was released after 30 min (Figure 12b).

As expected, the formulation composition has a decisive influence on the release of the active ingredient. Since bPMMA is a pH-dependent polymer that dissolves at a pH below 5.5, the release of enalapril from the bPMMA extrudates is prolonged at higher pH compared to the extrudates with the polymer SOL. However, a fast drug release from the extrudates with bPMMA in 0.1 N hydrochloric acid could be observed. Thus, the criterion of Q = 80% after 30 min could be fulfilled. Depending on the intended target profile, a suitable formulation has to be identified.

## 4. Conclusions

The obtained results showed that the peptidomimetic drug enalapril maleate could degrade during the hot-melt extrusion process at temperatures above 120 °C, more than 30 °C below the degradation temperature identified via thermo analysis. The main thermal degradation product formed during this process was the cyclization product enalapril diketopiperazine (Imp-D). Extrusion with bPMMA and SOL at 100 °C revealed a higher drug content in the formulation containing bPMMA. FT-IR data hints towards a cation-anion interaction with the basic bPMMA and enalapril maleate, which might have a stabilizing effect. Further formulation development and optimization of the process conditions during hot-melt extrusion could completely avoid the degradation of the drug enalapril maleate.

Our study demonstrates that by selecting suitable polymers and extrusion conditions, thermo-sensitive drugs can be hot-melt extruded and the formation of degradation products can be avoided. The question to what extent other even more thermo-sensitive compounds, such as proteins and peptides, can also be stabilized by melt extrusion remains to be clarified in the future. Furthermore, it should be noted that the formulation, in particular the polymers used, can have a decisive influence on the properties of the extrudate, such as the physical properties, the content as well as the release of the active ingredient. This work highlights the importance for formulation scientists to first know the properties of the active ingredient in order to select suitable polymers for melt extrusion.

## Figures and Tables

**Table 1:** Composition of HME formulations for screening experiments (*w*/*w*).

Formulation	Matrix (%)				Plasticizer (%)		Glidant (%)	
F1	K 12 PF	74.5			PEG 6.000	15	SiO_2_	0.5
F2	HPMC	74.5	bPMMA	10	PEG 6.000	5	SiO_2_	0.5
F3	K 12 PF	30	K VA 64	30	PEG 6.000	29.5	SiO_2_	0.5
F4	HPMC	84.5			PEG 6.000	5	SiO_2_	0.5
F5	bPMMA	44			PEO	44	SiO_2_	2.0
F6	SOL	44.75			PEO	44.75	SiO_2_	0.5

**Table 2:** Process parameters for HME for the different formulations.

Formulation	Powder Feed Rate (g/h)	Screw Speed (1/min)	Temperature (°C)
F1	50	50	130
F2	50	25	130
F3	50	25	140
F4	50	35	140
F5	100	35	150
F6	100	25	100

**Table 3:** Temperature profiles across the different zones of the extruder barrel (°C).

Formulation	Zone 1	2	3	4	5	6	7	8	Die
F5	20	20	100	100	100	100	100	100	100
F5	20	20	70	70	70	70	70	70	70
F6	20	20	100	100	100	100	100	100	100

**Table 4:** Gradient conditions for the separation of enalapril and its related substances.

Time [min]	Acetonitrile (% *v*/*v*)	Buffer (% *v*/*v*)
0–5.0	5	95
5.0–8.0	5 → 25	95 → 75
8.0–16.0	25	75
16.0–24.0	25 → 55	75 → 45
24.0–26.0	55	45
26.0–26.1	55 → 95	45 → 5
26.1–28.0	95	5
28.0–28.1	95 → 5	5 → 95
28.1–30.0	5	95

**Table 5:** Optimized gradient for the separation of enalapril and its related substances.

Time [min]	Acetonitrile (% *v*/*v*)	Buffer (% *v*/*v*)
0–1.0	2	98
1.0–1.2	2 → 25	98 → 75
1.2–5.0	25	75
5.0–7.5	25 → 40	75 → 60
7.5–9.0	40 → 75	60 → 25
9.0–11.0	75 → 95	25 → 5
11.0–12.5	95	5
12.5–12.6	95 → 2	5 → 98
12.6–15.0	2	98

**Table 6:** Particle size of raw materials (*n* = 3, mean ± SD).

Substance	x_10_ (µm)	x_50_ (µm)	x_90_ (µm)
Enalapril maleate	6.2 ± 0.1	47.2 ± 1.3	181 ± 6.9
bPMMA	3.8 ± 0.1	9.7 ± 0.1	37.3 ± 26.9
SOL	191 ± 4.6	308 ± 6.1	483 ± 5.6
PEO	14 ± 0.6	111 ± 8.6	320 ± 15.6

## Data Availability

The data presented in this study are available upon request from the corresponding author.

## Abbreviations

ACEI	angiotensin-converting enzyme inhibitor
Ala	alanine
BCS	Biopharmaceutics Classification System
bPMMA	basic butylated methacrylate copolymer
CRS	chemical reference standard
CV	coefficient of variation
DKP	diketopiperazine derivative
DSC	Dynamic Scanning Calorimetry
DTG	Derivative Thermogravimetric Analysis
EM	enalapril maleate
ENP	enalapril
FDM	Fused Deposition Modeling
FT-IR	Fourier transform-infrared spectroscopy
HME	hot melt extrusion
HPLC	High Performance Liquid Chromatography
HPMC	hydroxypropyl methyl cellulose, hypromellose
Imp	impurity
K	Kollidon
LOD	limit of detection
LOQ	limit of quantification
PF	Pyrogen-free
Phe	phenylalanine
Pro	proline
SD	standard deviation
SEM	Scanning Electron Microscopy
SiO_2_	fumed silica
SOL	Soluplus
Tg	glass transition temperature
TGA	thermogravimetric analysis
VA	poly(vinylpyrrolidone-vinyl acetate)-copolymer
WHO	World Health Organization
